# Maladaptive behaviors in disasters: case study evaluation of Hurricane Hugo, Hurricane Katrina and the Haiti Earthquake

**DOI:** 10.3389/fpubh.2024.1396517

**Published:** 2024-08-27

**Authors:** Andrew M. Milsten, Christopher S. Kang, Ira Nemeth

**Affiliations:** ^1^University of Massachusetts Chan Medical School, Emergency Medicine Department, Worcester, MA, United States; ^2^Madigan Army Medical Center, Tacoma, WA, United States; ^3^Baylor College of Medicine, Houston, TX, United States

**Keywords:** disaster myths, looting, hurricanes, earthquakes, Haiti 2010 earthquake, Hurricane Katrina, Hurricane Hugo, disaster psychology

## Abstract

Maladaptive behaviors during a disaster refer to actions that do not benefit the individual or society. Quarantelli highlights several maladaptive behaviors myths associated with disasters: widespread antisocial behavior, passivity, role conflict or abandonment, and sudden widespread mental health breakdowns (1). Despite early work reporting these myths, the common perception is that maladaptive behaviors such as rioting, looting, panic, and criminal conduct are prevalent in the wake of disasters. This is despite research by de Ville de Goyet and Arnold which has called on public officials and the media to stop propagating false disaster myths (2, 3). The classic academic response has been that this is a misconception and that, in fact, such behaviors are a very small part of the overall disaster and are mostly non-existent. Misconceptions about the prevalence of maladaptive behaviors can lead to inappropriate resource allocation, such as allocating extra police officers to prevent looting when the overall crime rate for the most part, decreases during disasters (4). Furthermore, while there are several persistent maladaptive behaviors myths, this is confounded by the presence of actual negative behaviors post disaster: false damage claims, insurance fraud, illegally obtaining relief supplies, failure to provide contracted repair services, hoarding of essential items, psychological trauma (which can lead to intergenerational transmission of the disaster memory) and medications and price gouging (5).When reading lay-press articles about recent disasters, it appears that these behaviors are on the rise. This raises the question: Has there been a change in the basic human reaction to disasters and are maladaptive behaviors on the rise? This review article focuses on case studies from three natural disasters: Hurricanes Hugo and Katrina, and the Haiti Earthquake. The goal of this review article is to evaluate these three natural disasters for evidence of maladaptive behaviors.

## Introduction

Maladaptive behaviors during a disaster refer to actions that do not benefit the individual or society. Quarantelli highlights several maladaptive behaviors myths associated with disasters: widespread antisocial behavior, passivity, role conflict or abandonment, and sudden widespread mental health breakdowns (1). Despite early work reporting these myths, the common perception is that maladaptive behaviors such as rioting, looting, panic, and criminal conduct are prevalent in the wake of disasters. This is despite research by de Ville de Goyet and Arnold which has called on public officials and the media to stop propagating false disaster myths (2, 3). The classic academic response has been that this is a misconception and that, in fact, such behaviors are a very small part of the overall disaster and are mostly non-existent. Misconceptions about the prevalence of maladaptive behaviors can lead to inappropriate resource allocation, such as allocating extra police officers to prevent looting when the overall crime rate for the most part, decreases during disasters (4). Furthermore, while there are several persistent maladaptive behaviors myths, this is confounded by the presence of actual negative behaviors post-disaster: false damage claims, insurance fraud, illegally obtaining relief supplies, failure to provide contracted repair services, hoarding of essential items, psychological trauma (which can lead to intergenerational transmission of the disaster memory) and medications and price gouging (5).

When reading lay-press articles about recent disasters, it appears that these behaviors are on the rise. This raises the question: Has there been a change in the basic human reaction to disasters and are maladaptive behaviors on the rise? This review article focuses on case studies from three natural disasters: Hurricanes Hugo and Katrina, and the Haiti Earthquake. The goal of this review article is to evaluate these three natural disasters for evidence of maladaptive behaviors, with our hypothesis being that these behaviors are still in the minority and are outweighed by post-disaster positive behaviors and growth. Our secondary hypothesis is that maladaptive behaviors only appear to be on the rise because of the media’s focus on sensational news topics that attract viewers, as these kinds of behaviors lend themselves to being dramatic news stories. Social media also plays a role in perpetuating disaster myths. Finally, this review article will focus only on the following post-disaster behaviors: looting and criminal behaviors.

## Methods

An extensive review of the available literature was conducted using the computerized databases Medline from 1977 through March 2024. The keywords “looting,” “panic,” “maladaptive behaviors,” “rioting” and “criminal conduct” were used as search terms. Articles were selected if they contained information pertaining to maladaptive behaviors after disasters. Furthermore, articles about Hurricanes Hugo and Katrina and the Haiti earthquake were searched and collected for analysis. Further review of the topic was done through web searches for lay-press articles using the same search terms. Selected articles were read, abstracted, analyzed, and compiled.

## Results

### St. Croix

Kuligowski points out that even though the media exaggerates most maladaptive behaviors, there are definite cases. A specific example is the looting after Hurricane Hugo hit St. Croix in 1989. Hurricane Hugo was an enormous storm affecting a large area (from North Carolina down through the Caribbean), but St. Croix was the only country to experience significant looting. The authors suggest that this may have been due to the extent to which St Croix was destroyed (>90% of homes and infrastructure), the ineffectual local officials and subsequent lack of hope that victims experienced, and the high rate of pre-storm crime and class inequality (6). Quarantelli also looked at looting in St Croix and postulated the same reasons as Kuligowski, with the addition of the St Croix police force being corrupt as an additional cause (7).

### Katrina

Barsky studied the looting that was also present after Hurricane Katrina and found several interesting phenomena. This included behaviors such as fear of looting leading to reluctance to evacuate. While the media’s dramatic portrayal of looting—which conveyed a picture of rampant and reckless looting—was inaccurate, it was noted that more looting occurred downtown and spiked when the city reopened. Businesses, not private homes, were looted and the authors reported that this might have been linked with past individual criminal behaviors and social inequalities. The authors make a distinction between need-based looting and luxury looting, with some anecdotal evidence pointing to more need-based looting around the first week after the storm (8).

How much crime occurred after Hurricane Katrina is subject to debate, but a full accounting is hampered because many basic functions of government were not operational during the crisis (for example, the 911 emergency telephone operators left their telephones when water began to rise around their building). Dwyer and Drew reported that New Orleans was gripped with the fear of crimes. These fears altered how public agencies responded to the disaster, how assets were deployed and how medical care was affected. In the end, though, most of the stories “were figments of frightened imaginations, the product of no reliable communications, and perhaps the residue of the raw relations between some police officers and members of the public” (9). Bailey examined the FBI’s annual Uniform Crime Report for violent crimes and found that the New Orleans murder rate dropped immediately after the hurricane, rose for 6 months, then decreased to pre-Katrina levels after July 2006 (10). Jacob et al. evaluated Hurricane Katrina behaviors through the lens of the social attachment model (11). They reported that most people responded positively and generously after Hurricane Katrina. The dominant response in community disasters was to seek contact with loved ones and familiar places, leading to increased altruism, camaraderie, and social solidarity rather than social breakdown.

### Haiti

A congressional report noted that in pre-earthquake Haiti (before 2010), poverty was already severe. Over one-half of the population (54%) was living on less than $1 a day; 78% live on $2 or less a day, according to the World Bank (12). Hunger was also widespread: 81% of the national population did not have access to the minimum daily ration of food defined by the World Health Organization. Furthermore, there were the elements of an unsound environment resulting from deforestation.

Regarding criminal behavior, such as looting, reports from Haiti convey a mixed picture. As indicated in the congressional report, security and military assets had to be diverted throughout the country, to control violence. Distribution of humanitarian aid was slowed down, partially due to damaged airports but also because this aid was turned away in favor of military support. Furthermore, there was criticism of the US for deploying military personnel on the one hand, and requests for more military (and police) aid on the other (13, 14). Attacks on aid deliveries led World Food Program trucks to be escorted with armed security escorts (15, 16). The level of unrest and violence did not go unnoticed by governmental organizations, which were trying to supply aid. Former US president Bill Clinton, the UN special envoy to Haiti, implored people to continue giving aid despite reports that some Haitians were angry and looting, indicating that these behaviors are short-lived phenomena and do not represent the entire country (16). Not all the looting during Haiti was motivated by hunger and thirst, some reports indicated that looting seemed to be partially controlled by vigilante justice and angry mobs (17).

Not all media reports from Haiti indicated violence. A 2010 reporting from General Hospital in Port-Au-Prince noted the absence of violence and that “reports of violence in the city have been overblown by the media and have affected the delivery of aid and medical services.” (18). Further reports of pro-community behavior included digging in the rubble to find survivors, sharing food, helping the injured and dignified burial ceremonies (19).

Romero and Lacey reported on this mixed picture of maladaptive and pro-community behavior in Haiti (15): “While most of this city of 3 million people focused on clearing the streets of debris and pulling bodies out of the rubble …there were pockets of violence and anarchy, reports of looting and ransacking, and at least one lynching of an accused looter as police officers stood aside. Both impulses—the riotous theft and the vigilante response—were borne of desperation, the lack of food and water as well as the absence of law and order. Given the conditions, it was even more remarkable that a spirit of cooperation and fortitude prevailed nearly everywhere else, as people joined together to carry corpses, erect shelters and share what food they could find.”

## Discussion

Looting is one of the maladaptive behaviors commonly perceived to be associated with events during and shortly after a disaster. The term “looting,” from the Hindi word *lūṭ or to plunder, was first used to describe the wartime seizure of valuable goods by an invading army. However, the definition of looting has devolved over time and now commonly refers to the illegal taking of property during an unlawful entry. Outside of wartime, the application of this contemporary description of looting to the study of and planning for disaster response has become problematic, especially when taken in the context of three distinctive factors.*

*The first factor is the inherent nature of the event, civil unrest or disaster* (6). The second factor is the demographics of the affected community, especially cultural and socioeconomic dynamics. The third factor is the magnitude of the disaster and its impact on the availability and proximity of assistance and key resources, especially necessities.

These three factors seem to influence the presence of post-disaster looting: pre-incident levels of social inequality, a certain but undefined level of pre-impact criminal behavior in society and inefficient and/or corrupt police force (both present in New Orleans and Katrina). So, to some degree, a level of pre-disaster criminal behavior may lead to looting and this behavior did not emerge due to the disaster but was already in place. This phenomenon was seen after the 2010 Chile earthquakes, where initial low levels of trust and social capital in Concepción led to looting and violence, as compared to regions with higher social capital that did not experience similar levels of antisocial behavior (20).

The looting that occurred after 2011 Japanese tsunami illustrates though, that the level of pre-disaster criminal behavior by itself, does not predict whether looting will occur. Japan is a country known for its low crime rate, yet still the population was concerned about disaster myths (including looting and panic) and there were reports of looting and cash robberies at home, convenience, and food stores (21–23). Nogami et al. took a deeper look at this spike in criminal behavior and found that public sources of disaster information (as opposed to private sources), such as television and Internet news websites, significantly influence the degree to which people believe in disaster myths. After the Great East Japan Disaster, the crime rate in the affected areas decreased by 18.2% compared to the previous year. The decrease was 15.4% in Iwate, 17.7% in Miyagi, and 20.0% in Fukushima, while the national crime rate dropped by 6.6%.

Early work on looting divided this behavior into that which occurs during civil disturbances versus natural disasters. Looting during civil disorders is widespread, but in natural disasters it is very rare. Furthermore, looting during civil disturbances usually involves local groups of residents from all segments of the population, while looting in natural disasters is more likely to be accomplished as a solitary act by community outsiders and may be a result, at least in part, of the notion that during a disaster people’s private property may be considered needed or available for the common good (4, 24). Gray and Wilson support the idea of natural disaster residential looting being done by outsiders in their study of two US disasters in the 1970’s (25).

Media can overplay maladaptive behaviors to the point that the coverage of the disaster’s destructive impact is replaced by images of people framed as looters or criminals. The media refers to the region as a war zone, in an effort to focus on dramatic behavior that will capture the audience’s attention and dollars. Tierney reported that media treatments reflect and reinforce broader societal and cultural trends. “Thus, myths concerning the panicky public, the dangers presented by looters, and the threat disaster victims pose to the social order serve to justify policy stances adopted by law enforcement entities and other institutions concerned with social control.” (6). The media put the victims into two oversimplified bins, bad people and helpless people. This overblown account of violence after Hurricane Katrina led to many unnecessary and costly actions such as deployment of extra security forces, setting up curfews that hinder victim assistance and fostered distrust between rescue personnel and the victims.

Disaster media reporting is often driven by dramatic events and, even if contradicted by evidence, the media continues to assume that looting is a common and newsworthy occurrence after a disaster (6). In 1998, H.W. Fischer noted that, “Looting is perhaps the most expected behavioral response to disaster. Both print and broadcast media personnel report on the alleged looting incidents, on steps being taken to prevent it…” (26). The absence of looting after some disasters was “often attributed to extraordinary security measures that have been taken rather than the fact that such behavior is inherently uncommon.” (27). This misattribution may lead to the further unnecessary deployment of security forces. During one of the most watched disasters in history, rampant looting was reported in New Orleans during Hurricane Katrina in 1995. Later that year, media analysts were “unanimous in their condemnation of how the media promoted myths of looting and violence in stories that were based almost entirely on rumor and hearsay.” (6). Numerous news outlets later admitted that some of “the reports of violence and crime were largely unsubstantiated” or “exaggerated” (9, 28).

The general public has long believed that looting occurs after disasters, because of media reporting, natural speculation, and a desire to protect personal property (27). After the 1952 Arkansas tornadoes, research interviews conducted by the National Opinion Research Center found that 58% of respondents in impacted areas and 52% of respondents in non-impacted areas had heard stories of looting, but that only 9% reported that they had lost property which they felt might have been looted (29). Similar discrepancies have been observed in multiple subsequent studies (25). Fear of looting was also widespread following Hurricanes Andrew and Katrina and may deter some people from evacuating their homes (30–32). Despite growing, albeit incomplete, research, the incidence of looting is less than what most of the public continues to believe (33).

### Limitations

*Identifying the actual incidence and prevalence of looting during and shortly after disasters has been problematic because of three issues*: the accuracy of police records, the accuracy of reporting by the media, and the perception of looting by the public. Investigating reports of looting may not be one of the higher priorities for law enforcement and the reports that are recorded by the police do not reflect the actual number of cases because some cases are never reported, erroneous, or classified as theft and not looting (8, 34). In some cases, businesses may claim missing inventory as losses from the disaster (35). Some cases may involve foraging for resources or the salvage of property by disaster victims and their friends and relatives (34, 36). This review article focused on only a few disasters and locations, which does not definitively inform international emergency mangers what to do in their specific circumstances nor does it push forward what we know in existing international literature on maladaptive behaviors in disasters. Lastly, only a handful of states formally classify looting as a formal crime distinct from theft and larceny (7, 37, 38).

## Conclusion

*Hypothesis* #1: Maladaptive behaviors are still in the minority and are outweighed by post-disaster positive behaviors and growth.

Currently, there is no definitive evidence of increased looting and panic after a natural disaster. If, however, the three factors mentioned above are present, a certain degree of looting should be expected. Whether this is need-based or luxury-based looting, cannot be determined from this review. Other contributing factors to consider *are the inherent nature of the event, civil unrest or disaster and* the magnitude of the disaster and its impact on the availability and proximity of assistance and key resources, especially basic necessities (6).

Another way to assess the probability of looting and panic after a disaster is to examine if the conditions producing those six factors (pre-incident levels of social inequality, a prior high level of criminal behavior in the society, and inefficient and/or corrupt police force and destructive nature of natural disasters or civil unrest) are likely to be more prevalent in the future. Arnold reviewed future hazards, vulnerabilities and disaster risks and noted several elements that have the potential to produce these six factors necessary for looting. These elements include increasing population growth and density (leading to worse economic imbalance, social inequality, urbanization and crime), global warming and environment degradation (producing more droughts and famine related disasters and more intense Hurricanes), and inadequate public health infrastructure and governmental regulations for over-crowded and disaster-prone populations (39).

Looting and other maladaptive behaviors will continue to occur after disasters and, thus, needs to be considered by public safety agencies and incorporated into disaster response policies. However, the levels and types of response may not be easy to determine. Several factors may assist the appropriate prioritization of responses to mitigate any looting that may occur after future disasters—inherent nature of the disaster, demographics of the affected community, and the magnitude of the event.

*Hypothesis* #2: Maladaptive behaviors only appear to be on the rise because of the media’s focus on sensational news topics that attract viewers, as these kinds of behaviors lend themselves to being dramatic news stories. Social media also plays a role in perpetuating disaster myths.

This review article has clearly highlighted that the media (including social media) often exaggerates the occurrence of maladaptive behaviors like looting and criminal activities during disasters and that sensational news stories attract more viewers, leading to a distorted perception that such behaviors are widespread. Dramatic media portrayals contribute to the belief that antisocial behaviors are common during disasters, despite research indicating they are relatively rare. Furthermore, this perception can influence public officials’ decisions, leading to misallocation of resources, such as deploying additional security forces unnecessarily. After a disaster, the media have an important role to play in recovery. They can both reflect and reinforce broader societal trends and stereotypes, not portray disaster victims as either perpetrators of violence or helpless victims and should strive for balanced reporting, focusing on both the challenges and the positive behaviors exhibited by communities during disasters.
